# Cerebellar Cathodal Transcranial Direct Stimulation and Performance on a Verb Generation Task: A Replication Study

**DOI:** 10.1155/2017/1254615

**Published:** 2017-02-14

**Authors:** K. Spielmann, R. van der Vliet, W. M. E. van de Sandt-Koenderman, M. A. Frens, G. M. Ribbers, R. W. Selles, S. van Vugt, J. N. van der Geest, P. Holland

**Affiliations:** ^1^Rijndam Rehabilitation Institute, P.O. Box 23181, 3001 KD Rotterdam, Netherlands; ^2^Department of Rehabilitation Medicine, Erasmus MC, University Medical Center Rotterdam, P.O. Box 2040, 3000 CA Rotterdam, Netherlands; ^3^Department of Neuroscience, Erasmus MC, University Medical Center Rotterdam, P.O. Box 2040, 3000 CA Rotterdam, Netherlands; ^4^Erasmus University College, Rotterdam, Netherlands; ^5^Department of Plastic and Reconstructive Surgery, Erasmus MC, University Medical Center Rotterdam, P.O. Box 2040, 3000 CA Rotterdam, Netherlands; ^6^Department of Biomedical Engineering, Ben-Gurion University of the Negev, Beer-Sheva, Israel

## Abstract

The role of the cerebellum in cognitive processing is increasingly recognized but still poorly understood. A recent study in this field applied cerebellar Transcranial Direct Current Stimulation (c-tDCS) to the right cerebellum to investigate the role of prefrontal-cerebellar loops in language aspects of cognition. Results showed that the improvement in participants' verbal response times on a verb generation task was facilitated immediately after cathodal c-tDCS, compared to anodal or sham c-tDCS. The primary aim of the present study is to replicate these findings and additionally to investigate possible longer term effects. A crossover within-subject design was used, comparing cathodal and sham c-tDCS. The experiment consisted of two visits with an interval of one week. Our results show no direct contribution of cathodal c-tDCS over the cerebellum to language task performance. However, one week later, the group receiving cathodal c-tDCS in the first visit show less improvement and increased variability in their verbal response times during the second visit, compared to the group receiving sham c-tDCS in the first visit. These findings suggest a potential negative effect of c-tDCS and warrant further investigation into long term effects of c-tDCS before undertaking clinical studies with poststroke patients with aphasia.

## 1. Introduction

Transcranial Direct Current Stimulation (tDCS) has become increasingly popular in neuroscience and neurorehabilitation. This user-friendly noninvasive form of brain stimulation can either increase or reduce neuronal excitability in a polarity-specific manner [1, 2]. Positive or anodal stimulation is proposed to increase activity in the brain area under the electrode whereas negative or cathodal stimulation would do the opposite. tDCS has been used for fundamental research to understand the functional organization of the brain and additionally it has been investigated in a clinical setting. Examples of such clinical studies include attempts to treat patients with poststroke aphasia or hemiplegia, Parkinson's disease, and depression [3–6]. However, despite a large body of tDCS literature reporting positive results, the reproducibility of these results is questioned [7, 8].

Recent studies have applied tDCS to understand the different functional domains of the cerebellum, a brain structure traditionally thought to be solely related to motor control but recently suggested to also be engaged in cognitive processes [9]. A role of the cerebellum in cognitive processing is supported by reports of cognitive deficits following injury to the cerebellum as well as anatomical and neuroimaging studies [10, 11]. Topographically, cerebellar lobules VI and VII were found to have projections to cortical association areas involved in cognitive processes [11]. Neuroimaging studies have shown that regions of lobule VII are involved in prefrontal-cerebellar loops [12–14]. Specifically, language processing and executive functioning activated regions of lobule VII [14]. Taken together, these studies demonstrate the role of prefrontal-cerebellar loops in cognitive processing; specifically, it has been suggested that the Purkinje cells in the right cerebellum have an inhibitory effect on the contralateral cortical prefrontal regions (i.e., cerebellocortical inhibition) [9, 11–14].

The efficacy of cerebellar tDCS (c-tDCS) in modulating cerebellocortical inhibition has previously been confirmed by Galea et al. [15]. They combined Transcranial Magnetic Stimulation (TMS) with c-tDCS and demonstrated that anodal c-tDCS to the right cerebellum increases the inhibitory effect to the primary motor cortex while cathodal c-tDCS to the right cerebellum reduces this effect. As Purkinje cells are the sole inhibitory output of the cerebellum, this observation suggests that anodal c-tDCS leads to increased activity of these neurons while cathodal c-tDCS leads to decreased activity. In addition, electrophysiological animal studies confirmed modulation of Purkinje cell activity with electrical stimulation [16, 17]. However, in humans, whether these changes in Purkinje cells firing are direct or depend on other cerebellar neurons is currently unknown. Given the highly homogenous anatomy of the cerebellar cortex, it would seem likely that c-tDCS affects the prefrontal cortex similarly to the motor cortex. This means anodal c-tDCS would decrease prefrontal cortex activity whereas cathodal c-tDCS would increase prefrontal cortex activity. However, literature regarding the efficacy of c-tDCS is inconsistent, for example, a study by Doeltgen et al. [18] reported that anodal c-tDCS may reduce the inhibitory effect on the primary motor cortex. Also, a study focusing on language functioning [19] found that both anodal and cathodal c-tDCS enhanced the performance on a phonemic fluency task.

An interesting recent study that investigated right cerebellar involvement in cognitive processing employed c-tDCS to study prefrontal-cerebellar loops in arithmetic and language aspects of working memory and attention [20]. Pope and Miall [20] hypothesized that cathodal c-tDCS over the right cerebellum lobule VII would reduce the inhibitory tone exerted by the Purkinje cells over prefrontal regions, causing disinhibition of the contralateral prefrontal regions. Disinhibition of prefrontal regions in turn could improve performance, especially on cognitively demanding tasks. Pope and Miall used arithmetic and language tasks with varying levels of cognitive demand and reported that the improvement in participants' verbal response times was facilitated by cathodal c-tDCS over the right cerebellum, compared to anodal or sham c-tDCS over the same region. Additionally, response times became less variable. As the improvement was greatest for the more cognitively demanding versions of the arithmetic and language task, the authors speculated that the cerebellum is capable of releasing cognitive resources by disinhibition of prefrontal regions, enhancing performance when tasks become cognitively demanding. Further support for this hypothesis was later found by demonstrating that stimulation of the prefrontal cortex with anodal tDCS achieves the same effect as cathodal c-tDCS, specifically for the task assessing arithmetic aspects [21].

In the present study, we were specifically interested in the potential improvement in language task performance after c-tDCS, as reported by Pope and Miall [20]. Right cerebellar involvement in language processing has been highlighted in several studies [22–24]. Further, a Positron Emission Tomographic (PET) study [25, 26] and a Functional Magnetic Resonance Imaging (fMRI) study [27] have demonstrated the involvement of left hemisphere areas and the right cerebellum during a verb generation task. The application of c-tDCS may contribute to our understanding of the prefrontal-cerebellar loops and language processing in healthy subjects but could also be interesting for future clinical applications [28]. Recent clinical studies applying cerebral tDCS in poststroke aphasia patients have already shown promising effects [29–31] and c-tDCS might possibly further contribute to the recovery of these patients. However, the results of cerebellar stimulation on language in healthy subjects await replication before translation to the clinical setting is justified.

The primary aim of the present study was to replicate the facilitatory effect immediately after cathodal c-tDCS on language task performance, as reported by Pope and Miall (i.e., their experiment 2) [20]. The task setup and outcome measures are similar to their study. In contrast to their between-subject design, the present study performed a crossover within-subject design, comparing cathodal and sham c-tDCS, in order to reduce the impact of individual variability in the response to tDCS [32]. The experiment consisted of two visits with an interval of one week; therefore, this design allowed us to investigate the long term effects of stimulation by measuring the same participants one week later.

## 2. Methods

### 2.1. Design

**Figure 1:**
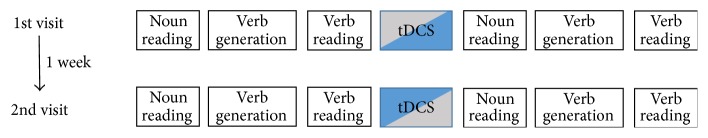
Study design: participants complete 2 visits with a one-week interval, receiving cathodal (blue) or sham c-tDCS (grey) in a counterbalanced order.

The present study used the same task described in experiment 2 of the study of Pope and Miall [20]. Their study had a double-blind between-subject design comparing anodal c-tDCS, cathodal c-tDCS, and sham c-tDCS (for further details, see [20]). The present study has a double-blind crossover within-subject design, comparing cathodal c-tDCS and sham c-tDCS (see Figure 1). The experiment consisted of two visits with an interval of one week. In each visit, a different stimulation condition (cathodal or sham c-tDCS) was applied and this order was counterbalanced among participants. Similar to the study of Pope and Miall, response accuracy and verbal response times were collected before and after cathodal c-tDCS and sham c-tDCS on three language tasks: noun reading, verb reading, and verb generation.

### 2.2. Sample Size Calculation

Power calculations were based on the reported effects of the study of Pope and Miall [20], specifically the interaction effect for verbal response times (Group × Block × Task, *F*(20,570) = 1.83 corresponding to a Cohen's *f* of 0.18) and the interaction effect for a computed variable “learning” (Session × Task × Group, *F*(1,114) = 4.50 corresponding to a Cohen's *f* of 0.28). For a study design with 4 repeated measurements (cathodal compared to sham; before tDCS compared to after tDCS), a within-patient correlation of 0.75, an alpha of 0.05, a power of 0.80, and a Cohen's *f* effect size of 0.18, we need 23 subjects. For a study design with 4 repeated measurements (cathodal compared to sham; before tDCS compared to after tDCS), a within-patient correlation of 0.75, an alpha of 0.05, a power of 0.80, and a Cohen's *f* effect size of 0.28, we need 11 subjects. Based on these power calculations, our aim was to include 24 subjects (in order to have an even number of subjects for the counterbalancing procedure).

### 2.3. Participants

Twenty-four healthy and native Dutch speakers (18 women, 6 men; age range 19–29 years, mean ± SD: 22 ± 2.36 years) with normal vision and normal speech (i.e., no stammer) were recruited from the Erasmus University Rotterdam for a small monetary reward. Exclusion criteria were left handedness and dyslexia. Right handedness was based on an Edinburgh Handedness Inventory score ≥ 50 [33], and the absence of dyslexia was self-reported. All participants gave informed consent and the study has been approved by the Medical Ethics Committee of the Erasmus MC, University Medical Center Rotterdam.

### 2.4. Tasks and Stimuli

We used the three language tasks that were used in the study of Pope and Miall [20]: a noun reading task, a verb generation task, and a verb reading task. For the reading tasks, participants have to read the presented noun or verb aloud as soon as it appeared on the computer screen. For the verb generation task, participants have to produce an appropriate verb as quickly as possible in response to the noun presented on the screen. For a Dutch version of these tasks, we prepared Dutch word lists including 40 nouns and 40 matched verbs. First, all nouns of the verb generation task used by Pope and Miall [20] were translated. Some of the nouns could not be translated into Dutch and some verb productions were strongly related to the morphological form of the item due to an identical wordstem (e.g.,* fiets, fietsen*, meaning “bike, biking”). The list of nouns was therefore supplemented by the set of Dutch nouns of De Witte et al. [34], resulting in a list of 124 concrete nouns related to manipulable tools and objects that were potential stimuli for the language experiment. The stimuli of the final word list were chosen on the basis of responses in a verb generation task from a pilot group (*n* = 22). Only noun-verb pairs generated by more than half of the pilot group were selected for the final word list. If two or more nouns elicited the same verb, these nouns were excluded. Also nouns eliciting nonaction verbs (e.g., “oven-bake”) were excluded. The final word list, including 40 nouns and 40 matched verbs, was split up in two lists (list A and list B): one list was presented before c-tDCS and the other after c-tDCS. The order of lists A and B was counterbalanced across participants. Specifically, during the first visit, half of the group was presented with list A before c-tDCS and list B after c-tDCS. During the second visit, this same group was presented with list B before c-tDCS and list A after c-tDCS. For the other half of the group, the order of presentation was reversed, thus starting during the first visit with list B before c-tDCS.

The stimuli were presented on a computer screen (48 cm × 28 cm) placed 65 cm in front of the participants. The tasks were designed and presented using MATLAB 2013a and Psychophysics Toolbox (version 3.0.12) [35, 36]. Each task comprised 6 blocks of 10 trials (i.e., 10 words) each. In the first five blocks, the same set of words was used but the order of the appearance of the words was randomized on a block-by-block basis. In the sixth block, a new set of words was presented, again in a randomized order. Each task lasted approximately 5 minutes. Participants had a break of at least 10 seconds between each task.

A microphone (model: Trust-MC 1200) was used to register the verbal response times. Each stimulus was replaced by the next stimulus when the microphone recorded a response. After a response was recorded, a black screen was displayed for 2 s before the next stimulus was presented.

### 2.5. Transcranial Direct Current Stimulation

Cathodal and sham c-tDCS were delivered through a pair of saline-soaked sponge electrodes (25 cm^2^ surface area) using a NeuroConn DC-stimulator. In the cathodal stimulation condition, participants received active stimulation of 2 mA for a duration of 20 minutes. Stimulation was automatically activated with a fade in of 30 s and after 20 minutes the stimulation was automatically deactivated with a fade out of 30 s. In the sham condition, participants received pseudo stimulation with a fade-in of 30 s and after 40 s the stimulation was automatically deactivated with a fade-out of 30 s. The average impedance was 23.7 ± 8.0 kΩ (mean ± SD) among participants. The cathode was placed over the right cerebellar cortex, 1 cm under and 4 cm lateral to the inion, which is defined as the location of the cerebellar lobule VII. The anode was placed over the right shoulder, that is, the right deltoid muscle [20].

### 2.6. Procedure

The experiment was performed inside a quiet cubicle. Participants performed the three tasks in the following order: noun reading, verb generation, and verb reading. For the reading tasks, participants were instructed to read the presented noun or verb aloud as soon as it appeared on the computer screen. For the verb generation task, they were instructed to produce an appropriate verb as quickly as possible in response to the noun presented on the screen. It was explained that an appropriate verb could be a verb that described what the presented noun may do or what it may be used for. It was emphasized that only one verb was to be produced. At the beginning of each task, one example was given and three test items were presented, which were items other than those in the experiment. For all tasks, responses were checked for accuracy by the researcher. All verbs produced during the verb generation task were written down by the researcher.

After completion of the three tasks, 20 minutes of cathodal or sham c-tDCS was applied. The electrodes were placed by the researcher. Both the researcher and the participant were blinded for stimulation condition, which was achieved by using two 5-number codes that can be entered into the tDCS device. These 5-number codes are provided by the manufacturer of the tDCS device. One code is related to start the real tDCS stimulation condition and the other code is related to start sham tDCS. A researcher of our research team (JG), who was not involved in the assessment of the experiment, provided these two 5-number codes. During the 20 minutes cathodal or sham c-tDCS, participants were instructed to look at a black computer screen. After the stimulation, participants performed the three tasks for the second time using parallel versions of word lists. In total, the experiment lasted approximately 90 minutes. After one week, each participant took the experiment for the second time, in which the other stimulation condition was applied. Next to that, the word list previously presented after c-tDCS was now presented prior to c-tDCS.

### 2.7. Statistical Analysis

Incorrect responses, missed responses, and outliers were removed before analysis. For the noun reading and the verb reading tasks, no incorrect responses were detected. For the verb generation task, nonwords, multiple word responses and responses that were not representative for what the noun may do or what it may be used for (e.g., “eyebrow–drawing”), were considered incorrect and were not included in the analysis. For each task, voice onset times were corrected manually from digital recordings if lip movement, swallowing, and heavy breathing were prior to the verbal response, because this influenced the microphone recording. Outliers, responses exceeding more or less than 2 standard deviations from the mean of that task, were removed. Specifically, the mean and standard deviation of all subjects responses per task determined the outlier levels.

Although we used test items, a novelty effect was found for the first trials (i.e., first word presented) of each block, shown by a larger reaction time. Because the mean for each block consisting of 10 trials was calculated, we decided to exclude the first trial in order to get a representative mean of the data. Further, in case of violations of sphericity, a Greenhouse-Geisser correction was applied and adjusted degrees of freedom are reported in the text.

In line with the study of Pope and Miall, the present study analyzed the data in terms of the mean and variability of verbal response times. Mean verbal response times for each block per task were analyzed with a repeated measures analysis of variance (ANOVA), using four factors. These factors are Condition (cathodal tDCS and sham), Session (pre-tDCS and post-tDCS), Task (noun reading, verb generation, and verb reading), and Block (six blocks per task). The variability of verbal response times between the three tasks and six blocks per task was analyzed with pairwise comparisons; a Bonferroni correction was used. The level of significance was set at *α* = 0.05. For the response variability, an ANOVA was performed on the within block standard deviations of the verbal response times across Block, Task, and Session and averaged by Condition.

Also in line with the study of Pope and Miall, the present study analyzed the data by computing the variables “learning” and “total learning variability.” The learning variable was computed by subtracting Block 5 from Block 1 and putting this as a variable in an ANOVA with Task × Session × Condition. For the total learning variability, the standard deviations of the learning variable (Block 5 − Block 1) across Task and Session and averaged by Condition were entered into an ANOVA.

The present within-subject design allows us to investigate the long term effects of stimulation by measuring the same subjects a week later. We therefore also performed an ANOVA including the between-subject factor visit-order. This between-subject factor indicates whether a participant received cathodal c-tDCS or sham c-tDCS at the first visit.

## 3. Results

In general, results are reported in the same way as in the study of Pope and Miall [20].  Table 1 presents an overview of the statistical results for the 4 variables that were analyzed: mean verbal response times, verbal response variability, learning, and total learning variability.  Table 1 only includes the factors and interactions that were reported as (near) significant in the study of Pope and Miall and will be explained further in the following paragraphs. Values are reported as mean ± standard error of the mean in the text unless otherwise specified.

### 3.1. Response Accuracy and Outliers

Participants made very few incorrect responses (1.9%) and very few missed responses (0.5%) were obtained. With regard to outliers, 3.5% of the responses were classified as outliers. The incorrect and missed responses and the outliers were excluded from further analysis.

### 3.2. Verbal Response Times

**Figure 2:**
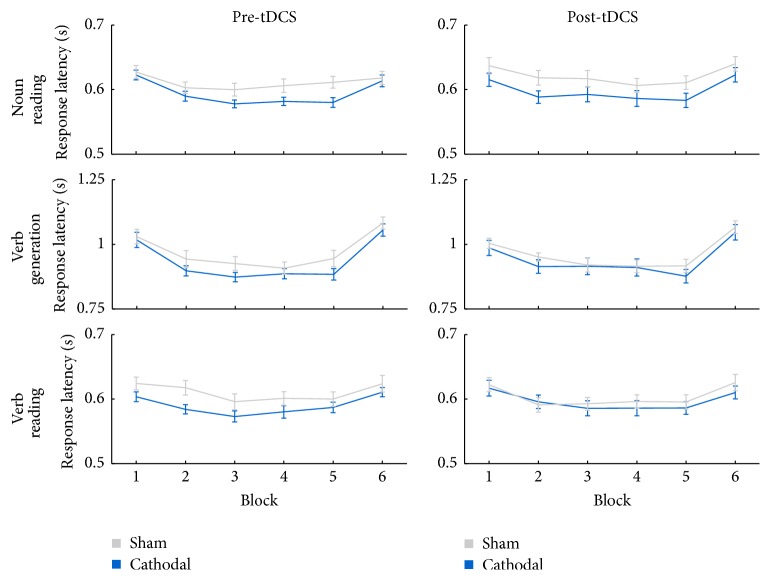
Results for the verbal response times (s), before and after tDCS, for each task and across the 6 blocks. Error bars represent the standard error of the mean (SEM).

Figure 2 presents the results of the verbal response times for each task and across the 6 blocks, before and after tDCS. In general, the range of verbal response times of the present study (0.573 s–1.082 s) was higher than the study of Pope and Miall [20]. A Condition × Task × Session × Block ANOVA revealed a large main effect (see Table 1) of Condition, with larger verbal response times in the sham condition (0.730 ± 0.011 s) compared to the cathodal condition (0.709 ± 0.010 s). However, there was no main effect of Session and no interaction effect of Condition × Session, therefore indicating no overall effect of tDCS on verbal response times.

In line with the study of Pope and Miall, a large main effect of Task was found, with larger verbal response times on the verb generation task (0.953 ± 0.016 s) compared to the noun reading (0.606 ± 0.007 s) and verb reading (0.600 ± 0.008 s) task. Also in line with Pope and Miall, a large main effect of Block was found. This can be described as a priming effect for block 1–5, meaning that the verbal response times are reduced across blocks 1–5 because the same words are repeated, and a novelty effect from block 5 to block 6, meaning an increase in verbal response time because new words are presented. The priming effect and the novelty effect were greater for the verb generation task, as shown by a large Task × Block interaction. Specifically, the verbal response times across blocks 1–5 were reduced more during verb generation than during noun reading and verb reading. The increase in verbal response times from block 5 to block 6 was greater for verb generation than for noun reading and verb reading.

### 3.3. Response Variability

For the response variability, a Condition × Task × Session × Block ANOVA revealed no main effect of Condition. A large main effect of Session was found, such that the response variability was greater after (0.096 ± 0.002 s) than before (0.091 ± 0.002 s) tDCS. However, there was no Condition × Session interaction, indicating no overall effect of tDCS on verbal response variability. In line with the study of Pope and Miall, there was a large main effect of Task, such that verbal response times were more variable during verb generation (0.168 ± 0.004 s) than during noun reading (0.054 ± 0.002 s) and verb reading (0.059 ± 0.002 s). Also, in line with Pope and Miall, a large main effect of Block was found, where response variability decreased across the 5 blocks of repeated words (i.e., priming effect) and then increased in block 6, when new word lists were shown (i.e., novelty effect). This pattern for the priming effect and the novelty effect was greater for the verb generation task, as shown by a large Task × Block interaction. Specifically, the response variability across blocks 1–5 was reduced more during verb generation compared to noun reading and verb reading. The increase in response variability from block 5 to block 6 was greater for verb generation than for noun reading and verb reading.

### 3.4. Learning

**Figure 3:**
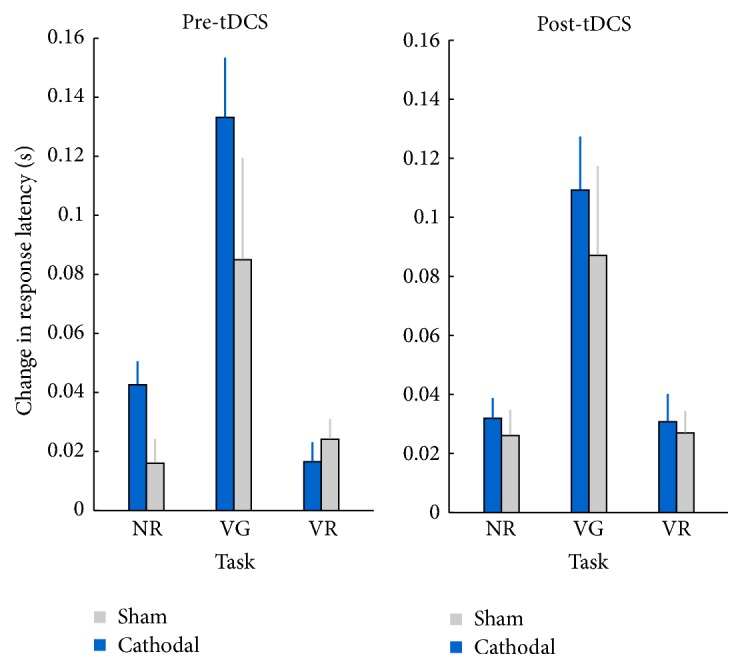
Results for the learning variable, calculated by subtracting the verbal response times (s) in block 5 from the verbal response times (s) in block 1. This difference is presented for each task, before and after tDCS. Error bars represent the standard error of the mean (SEM).

The results for learning, as reflected in the difference in response times between block 1 and block 5, are presented in Figure 3. A Condition × Task × Session ANOVA revealed no significant main effect of Condition and no significant main effect of Session, indicating there was no effect of tDCS. In line with the study of Pope and Miall, there was a large main effect of Task, such that there was a larger improvement of verbal response times across blocks 1–5 for the verb generation task (0.104 ± 0.015 s), compared to noun reading (0.029 ± 0.005 s) and verb reading (0.025 ± 0.004 s). In contrast with the study of Pope and Miall, the present study did not demonstrate a Condition × Session × Task interaction.

### 3.5. Change in Variability

For the total learning variability across blocks 1 to 5 (i.e., analyzing the standard deviations for the learning variable), a Condition × Task × Session ANOVA revealed no main effect of Condition. A large main effect of Session was found, such that the change in response variability was greater after (0.023 ± 0.004 s) than before (0.008 ± 0.005 s) tDCS. However, there was no Condition × Session interaction, indicating no overall effect of tDCS on the change in variability. In line with the study of Pope and Miall, there was a large main effect of Task, such that the change in response variability between blocks 1 and 5 was greater for verb generation (0.035 ± 0.011 s), than for noun reading (0.005 ± 0.002 s) and verb reading (0.006 ± 0.002 s). A significant, large Task × Session interaction was found, such that the change in response variability before and after tDCS was greater for the verb generation task, than for noun reading and verb reading. In contrast with the study of Pope and Miall, the present study did not demonstrate a Condition × Session × Task interaction.

### 3.6. Long Term Effects

#### 3.6.1. Verbal Response Times

**Figure 4:**
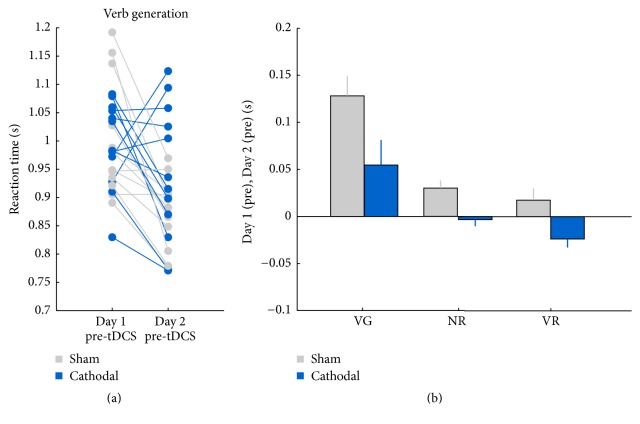
Results for the long term effects. (a) The individual verbal response times on the verb generation task, for visit 1 and visit 2. (b) The mean verbal response times for each task, subtracting performance in the second visit from the first visit. Error bars represent the standard error of the mean (SEM).

A Condition × Task × Session × Block ANOVA including blocks 1–5 and with visit-order as a between-subject factor (i.e., labeled as Visit) revealed a significant Condition × Visit interaction, *F*(1,22) = 8.362, *p* = 0.008, *η*^2^ = 0.275, such that the mean verbal response times showed a greater reduction for the group receiving sham in the first visit (first visit: 0.727 ± 0.016 s; second visit: 0.681 ± 0.014 s), than for the group receiving cathodal stimulation in the first visit (first visit: 0.717 ± 0.014 s; second visit: 0.715 ± 0.016 s). This effect was greater for the verb generation task, as shown by a Condition × Task × visit interaction, *F*(1.294,28.470) = 25.266, *p* < 0.001, *η*^2^ = 0.535.  Figure 4 presents this interaction effect, showing the mean verbal response times for each task and stimulation condition and comparing the first visit with the second visit. Specifically, the verbal response times for the verb generation task reduced more for the group receiving sham in the first visit (first visit: 0.963 ± 0.028 s; second visit: 0.864 ± 0.024 s) than for the group receiving cathodal first (first visit: 0.967 ± 0.024 s; second visit: 0.928 ± 0.028 s).

In line with the immediate c-tDCS results, the long term analysis shows a priming effect across blocks 1–5. Specifically, there was a Condition × Block × Visit interaction, *F*(4,88) = 3.026, *p* = 0.022, *η*^2^ = 0.121, such that the verbal response times across blocks 1–5 reduced more for the group receiving sham the first time.

#### 3.6.2. Response Variability

For the response variability, the ANOVA analysis also revealed a large interaction of Condition × Visit, *F*(1,22) = 14.274, *p* = 0.001, *η*^2^ = 0.394, such that the response variability reduced more for the group receiving sham the first time (first visit: 0.094 ± 0.004 s; second visit: 0.082 ± 0.003 s) than for the group receiving cathodal tDCS in the first visit (first visit: 0.096 ± 0.003 s; second visit: 0.089 ± 0.004 s). This effect was also more present for the verb generation task, as shown by a large, interaction effect of Stimulation × Task × Visit, *F*(1.558,34.280) = 40.123, *p* < 0.001, *η*^2^ = 0.646. Specifically, the response variability for the verb generation task reduced more for the group receiving sham the first time (first visit: 0.171 ± 0.009 s; second visit: 0.132 ± 0.007 s) than for the group receiving cathodal the first time (first visit: 0.186 ± 0.007 s; second visit: 0.152 ± 0.009 s).

In line with the immediate c-tDCS results, the long term analysis for the response variability also shows a priming effect across blocks 1–5. Specifically, there was a significant interaction effect of Condition × Block × Visit, *F*(4,88) = 2.596, *p* = 0.042, *η*^2^ = 0.106, such that the response variability across blocks 1–5 reduced more for the group receiving sham the first time. Finally, there was a significant interaction effect of Condition × Task × Block × Visit, *F*(3.728,82.018) = 4.302, *p* = 0.004, *η*^2^ = 0.164, such that, for the verb generation task, response variability across blocks 1–5 reduced more for the group receiving sham the first time.

#### 3.6.3. Post Hoc Tests: Additional Analysis of the Long Term Effects

**Figure 5:**
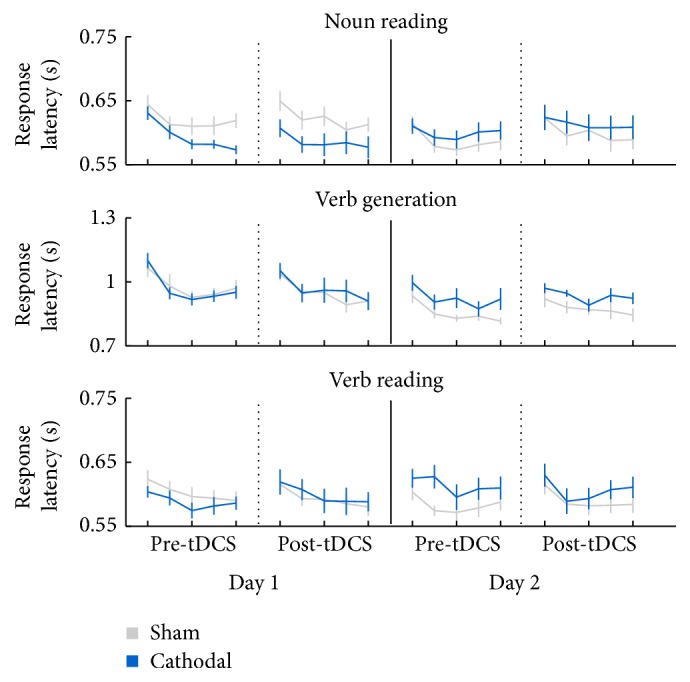
Verbal responses times (s) across blocks 1–5 and for each task, for the time points pre-tDCS visit 1, post-tDCS visit 1, pre-tDCS visit 2, and post-tDCS visit 2. Blue represents the group starting with the cathodal condition in the first visit and grey represents the group starting with the sham condition in the first visit. Error bars represent the standard error of the mean (SEM).

To further study the performance over time and the effect of visit-order, we have performed some additional analysis.  Figure 5 presents the performance over time, for each task and across blocks 1–5, for the time points before tDCS visit 1 (pre-tDCS visit 1), after tDCS visit 1 (post-tDCS visit 1), before tDCS visit 2 (pre-tDCS visit 2), and after tDCS visit 2 (post-tDCS visit 2). Blue represents the group starting with the cathodal condition in the first visit and grey represents the group starting with the sham condition in the first visit.

We studied specifically the performance from time point post-tDCS visit 1 to the time point pre-tDCS visit 2 in order to analyze whether performance improved between visits (i.e., offline learning). Also, the same set of words was under examination for these 2 time points. An ANOVA including these time points, with visit-order as the between-subject variable, revealed that the average performance across blocks 1–5 improves from post-tDCS visit 1 (0.719 ± 0.014 s) to the pre-tDCS visit 2 (0.693 ± 0.012 s), shown by a large effect, *F*(1,22) = 9.716, *p* = 0.005, *η*^2^ = 0.306. This effect could be interpreted as an effect of offline learning, so participants become better in a task after a time interval. Furthermore, the group receiving sham the first time improves more for these time points (0.721 ± 0.020 s in visit 1 compared to 0.674 ± 0.016 s in visit 2) than the group receiving cathodal tDCS the first time (0.717 ± 0.020 s in visit 1 compared to 0.712 ± 0.016 s in visit 2). This was shown by a large Stimulation × Visit interaction effect, *F*(1,22) = 6,467, *p* = 0.019, *η*^2^ = 0.227. However, these results include only the mean of all blocks, and so it is not possible to discern if any improvements in performance are a result of continued practice or if in fact performance has improved between visits (i.e., offline learning). Therefore, a further step in our analysis was to specifically analyze the time point post-tDCS block 5 of visit 1 and time point pre-tDCS block 1 of visit 2. An ANOVA including these time points, with visit-order as the between-subject variable, revealed that the performance on post-tDCS block 5 in visit 1 (0.696 ± 0.015 s) actually decreased in the pre-tDCS block 1 in visit 2 (0.730 ± 0.012 s). This was shown by a large effect of visit, *F*(1,22) = 9,190, *p* = 0.006, *η*^2^ = 0.295. Therefore, these data show no evidence for offline learning.

## 4. Discussion

The aim of the present study was to replicate the results of Pope and Miall by demonstrating that cathodal stimulation of the right cerebellum improves task performance on a verb generation task [20]. The task setup and outcome measures were similar to their study. Based on their results, showing a facilitatory effect immediately after cathodal c-tDCS, we compared cathodal c-tDCS and sham stimulation. In contrast with the between-subject design study of Pope and Miall, the present study used a crossover within-subject design, in order to reduce the impact of individual variability [32]. Participants had to complete two visits, with half of the group receiving cathodal c-tDCS the first time and half of the group receiving sham c-tDCS the first time. Our results did not show a facilitating effect of cathodal c-tDCS on verb generation, in terms of either verbal response times or variability. In line with Pope and Miall, the verbal response times were larger for the verb generation task, compared to noun reading and verb reading. This effect can be explained with the idea that the verb generation task requires lexical search processes and verbal response selection, while noun and verb reading requires only reading processes. Interestingly, the verbal response times on our tasks were longer than those reported by the original study. These longer reaction times could be due to linguistic factors of the words [37], for example, word length; that is, words with more phonemes need more time to process [38]. Indeed, on average, the words in our word lists were longer (mean ± SD: 6.13 ± 2.188 phonemes) than the lists of Pope and Miall (mean ± SD: 4.77 ± 1.376 phonemes) [20]. Further, in line with Pope and Miall, there was a reduction in response time across blocks 1–5 (i.e., priming effect) and an increase in block 6 (i.e., novelty effect).

The data of the present study do not confirm that cathodal c-tDCS over the right cerebellum lobule VII leads to disinhibition of the contralateral prefrontal regions and therefore to an improved performance on a cognitive demanding task (i.e., verb generation task). Previous studies have suggested that the Purkinje cells in the right cerebellum would have an inhibitory effect on the contralateral cortical prefrontal regions (i.e., cerebellocortical inhibition) [9, 11–14]. For language processing, right cerebellar involvement has also been suggested [22–24]. Specifically, for the verb generation task, a PET scan study and an fMRI study showed that the contralateral cerebellar hemisphere was actively involved [25–27]. However, when investigating the efficacy of c-tDCS in modulating cerebellocortical inhibition, motor-related studies demonstrate inconsistent findings. For example, one study demonstrates that anodal tDCS to the right cerebellum increases the inhibitory effect to the primary motor cortex while cathodal tDCS to the right cerebellum reduces this effect [15]. In contrast, another study in this field reports that anodal c-tDCS may reduce the inhibitory effect to the primary motor cortex [18].

Furthermore, the idea that the cerebellum constrains cortical activity which can be disinhibited by cathodal c-tDCS is also not consistently supported by cognition-related tDCS studies. For example, studies show contradictive results with regard to the application of tDCS to the right cerebellum and its effects on the performance on a verbal working memory (WM) task, that is, forward and backward digit span task. One study shows that cathodal c-tDCS leads to reduced forward digit span and blocks the practice dependent increase in backward digit span [39], while another study [40] shows that both anodal and cathodal tDCS impair practice dependent improvement in reaction times in a WM task. Further, Turkeltaub et al. [19] found that both anodal and cathodal c-tDCS enhanced the performance on a phonemic fluency task; however, the anodal effect was found to be more robust. Taken together, it seems that c-tDCS studies are not yet consistent whether anodal or cathodal c-tDCS improves or disrupts task performance in healthy subjects. Future studies need to further explore the specific polarity effects of c-tDCS in order to understand its usage for cerebellar dependent cognitive processing.

Interestingly, we observe a long term effect of c-tDCS in our data. When analyzing the data further by taking into account visit-order, we found that the group receiving cathodal c-tDCS the first time demonstrated poorer performance in the second visit in comparison to those who received sham stimulation the first time. First of all, the group receiving cathodal c-tDCS in the first visit demonstrate less improvement from visit 1 to visit 2. Also, the group receiving cathodal c-tDCS in the first visit show less improvement during the second visit (i.e., performance across blocks 1–5) compared to the group receiving sham the first time. Regarding response variability, the same findings are found; thus, the group receiving cathodal c-tDCS in the first visit show increased variability in verbal response times in the second visit and during the second visit (i.e., increased variability across blocks 1–5). In motor-related studies, this long term effect is often called a consolidation effect, meaning that after acquisition performance can become resistant to decay [41]. To our knowledge, studies investigating consolidation effects of c-tDCS on a language task are scarce, whereas there are several motor-related c-tDCS studies that investigate the effect of c-tDCS on a longer time scale. For example, one such study demonstrated that anodal c-tDCS would enhance general motor skill learning and sequence-specific learning, 35 minutes after tDCS stimulation [42]. Another study shows that anodal c-tDCS to the right cerebellum improves task performance on a temporal motor task in the follow-up tests (90 minutes and 24 h after training) [43]. Furthermore, a recent study provides evidence that cathodal c-tDCS impairs overnight retention of a force field reaching task [44]. Therefore, these motor-related studies show that, on a longer time scale, anodal c-tDCS may enhance performance, while cathodal c-tDCS may impair performance, which is in line with the long term results of the present study.

Studies focusing on the adaptation of movements and tDCS have demonstrated a dissociation between the acquisition phase and the consolidation phase [45, 46]. Specifically, anodal tDCS to the right cerebellum leads to an increased acquisition of new internal models whereas anodal tDCS to the motor cortex leads to improved consolidation. Therefore, the cerebellum is believed to rapidly acquire new internal models that are also quickly forgotten whereas the motor cortex learns more slowly but retains better (i.e., consolidation). A similar transfer of learning from the cerebellar cortex to other structures has been proposed for other cerebellar dependent adaptation tasks such as eye-blink conditioning or adaptation of the vestibule-ocular reflex [47]. In the present study, it is possible that these two partially separable effects are at work: short terms changes in firing rate of the cerebellum and additional effects on plasticity. First, cathodal c-tDCS may indeed reduce the firing rate of Purkinje cells and the inhibitory tone on the prefrontal cortex and therefore improve performance in tasks relying on these cortical areas, as found in the study of Pope and Miall. However, it should be noted that there is no direct neurophysiological evidence for this effect of c-tDCS specifically on the prefrontal cortex. Secondly, cathodal c-tDCS may also reduce plasticity in the cerebellar cortex and therefore retard the rate of learning there, subsequently reducing the amount that can be transferred to other areas for consolidation, which may be in line with the results of the present study.

The present within-subject design with several time points allows us to evaluate different subconcepts of consolidation. Consolidation can be described in terms of offline learning, that is, improvements in performance between visits, and memory stabilization, that is, reduced performance compared to the end of the previous visit but increased performance in comparison to the naïve state [48]. However, the degree to which either or both of these are possible is dependent on task structure and the particular skill under consideration. An important consideration in interpreting our results is separating the effect of repeated practice from true offline learning. The results of the present study show that the average performance across blocks 1–5 improves from time point post-tDCS in the first visit to time point pre-tDCS in the second visit. Furthermore, the group receiving sham the first time improves more for these time points than the group receiving cathodal stimulation the first time. Therefore, these results may show an effect of offline learning; however, if only the mean of all blocks is used as a measure of performance, it is not possible to discern if any improvements are a result of continued practice or if in fact performance has improved between visits [48]. Further analysis demonstrates that performance in both groups (i.e., the group receiving cathodal stimulation the first time and the group receiving sham the first time) decreased between block 5 of the first visit and block 1 of the next, despite the fact that the same set of words was under examination. These data therefore show no evidence for offline learning but that may be due to the relatively long period of time between visits or because this particular task is not appropriate for such changes. In the future, it will be interesting to test subjects again after a shorter interval to assay if offline learning is indeed possible with this task. It is important to note that offline learning has been investigated in an fMRI learning paradigm in which subjects had to learn a new lexicon and were tested 20 minutes later [49]. The degree of offline learning was positively correlated with the level of activation of the right cerebellum. Therefore, these data provide evidence for a role of the cerebellum in consolidation of a learning task that includes language/linguistic aspects. The differences between learning a new lexicon and learning associations within a known lexicon (as here), especially when concerning the cerebellum, are unknown and it is vital for proper delineation of tDCS effects that the specific task demands are well understood.

### 4.1. Limitations of the Study

First of all, it should be noted that the design of the present study with 1 week between 2 visits could interfere with replication of the original immediate effect reported by Pope and Miall. This interference could be due to effects of retesting the same words or a ceiling effect. Furthermore, in the present study, the subjects had one block of novel words at the end of the five blocks of repeated words which may have also acted as an interfering factor. As the majority of the results found in both the present study and the original Pope and Miall study can be found within blocks 1–5, it would be interesting to repeat the experiment with the omission of the novel words in block 6 to test if any interference is occurring. Finally, it should be noted that the majority of (c-)tDCS studies are described in the context of motor tasks and we therefore used these studies in order to interpret our results; however, the analogy between motor learning, consolidation, and the type of results presented here may be stretched.

### 4.2. Conclusion and Future Recommendations

The present study shows that long term effects of c-tDCS need to be taken into account when investigating the effect of c-tDCS on language task performance. Most tDCS studies with a motor or nonmotor learning task focus on direct results rather than long term learning effects (i.e., consolidation). Our findings warrant further investigation into long term effects of c-tDCS, to better capture its effect and how we can use this application to understand the complex role of the cerebellum on cognitive/language processing. Therefore, we first need to understand c-tDCS in healthy subjects, before undertaking clinical studies with poststroke patients with aphasia. To further explore the long term effect of c-tDCS on a cognitive language task, we would suggest to combine the design of Pope and Miall with the design of the present study. This combined design would describe the effect of c-tDCS in 3 conditions, anodal c-tDCS, cathodal c-tDCS, and sham (between-subject), and participants need to come twice in each condition (within-subject). This design allows us to evaluate the effect of anodal c-tDCS compared to the effect of cathodal c-tDCS, on a longer time scale. Furthermore, techniques such as EEG may be used to explore the effect of cerebellar tDCS and its polarity-specific effects on ongoing or induced activity in areas of the cortex associated with language.

## Figures and Tables

**Table 1:** Results of the study: verbal response time, response variability, learning, and learning variability.

Variable	Effect	df	*F*	*p*	*η* ^2^
Verbal response time	Condition	1, 23	4.81	0.039	0.173
	Task	1.16, 26.71	808.98	<0.001	0.972
	Block	5, 115	121.63	<0.001	0.841
	Task × Block	4.22, 97.15	37.16	<0.001	0.618
	Session	1, 23	0.10	0.750	0.004
	Task × Session	1.38, 1.20	0.77	0.427	0.032
	Condition × Task × Block	4.33, 99.63	0.77	0.558	0.032

Response variability	Session	1, 23	6.49	0.018	0.220
	Task	1.19, 27.37	655.93	<0.001	0.966
	Block	5, 115	17.63	<0.001	0.434
	Task × Block	4.31, 99.12	8.65	<0.001	0.273
	Condition × Block	5, 115	0.62	0.689	0.026
	Condition × Task × Block	4.00, 91.96	1.42	0.233	0.058

Learning	Task	1.20, 27.52	21.76	<0.001	0.486
	Task × Session	1.22, 27.96	0.47	0.537	0.020
	Task × Condition	1.18, 27.11	1.48	0.240	0.060
	Session × Condition	1, 23	0.36	0.555	0.015
	Session × Task × Condition	1.27, 29.10	0.35	0.608	0.015

Learning variability	Session	1, 23	5.45	0.029	0.192
	Task	1.09, 25.00	6.66	0.014	0.225
	Condition	1, 23	0.63	0.435	0.027
	Task × Session	1.24, 28.44	7.09	0.009	0.236
	Task × Condition	1.17, 26.84	0.34	0.600	0.014
	Session × Condition	1, 23	0.70	0.411	0.030
	Session × Task × Condition	1.06, 24.34	0.44	0.524	0.019
